# CraniofacialMorphometric Associations with Frontal Sinus Hypoplasia/Aplasia in Adults: Orbital and Upper Facial Differences on CT

**DOI:** 10.3390/diagnostics16132056

**Published:** 2026-06-30

**Authors:** Rezarta Taga Senirli, Nuriye Oz, Merve Yıldırım, Buket Yagci, Nigar Keles, Özer Erdem Gur

**Affiliations:** 1 Department of Otolaryngology, Antalya Training and Research Hospital, 07100 Antalya, Turkey; erdemkaptan@yahoo.com; 2 Department of Anatomy, Faculty of Medicine, Akdeniz University, 07058 Antalya, Turkey; nuriyeoz@akdeniz.edu.tr (N.O.); merve1991onder@gmail.com (M.Y.); nigarc@akdeniz.edu.tr (N.K.); 3 Department of Radiology, Antalya Training and Research Hospital, 07100 Antalya, Turkey; buketyagci@hotmail.com

**Keywords:** aplasia, facial morphometry, frontal sinus, hypoplasia, maxillary sinus

## Abstract

**Background/Objectives:** To evaluate whether frontal and maxillary sinus hypoplasia/aplasia are associated with differences in CT-based craniofacial morphometric measurements in adults. **Methods:** This retrospective case–control study included adults who presented to the otolaryngology outpatient clinic and underwent paranasal sinus CT at a single institution between 3 April 2023, and 30 May 2024. Of 3000 CT scans reviewed, 117 adults with frontal and/or maxillary sinus hypoplasia/aplasia and 53 healthy controls met the eligibility criteria. The sinus variation groups included unilateral frontal sinus variation (UFSV, *n* = 46), unilateral maxillary sinus variation (UMSV, *n* = 13), and bilateral frontal sinus variation (BFSV, *n* = 55); and bilateral maxillary sinus variation (BMSV, *n* = 3), which was described descriptively but excluded from the main statistical comparisons because of the very small subgroup size. Craniofacial morphometric distances were measured using standardized anthropometric landmarks, and group comparisons were performed using analysis of covariance adjusted for age and sex. **Results:** Significant adjusted between-group differences were found for left orbital breadth (*p* = 0.0001), left orbital height (*p* = 0.0250), right orbital breadth (*p* < 0.0001), biorbital breadth (*p* < 0.0001), upper facial breadth (*p* = 0.0204), and bizygomatic breadth (*p* = 0.0026). In general, the UFSV and BFSV groups showed lower adjusted values for orbital and upper facial measurements, whereas the healthy control and UMSV groups showed relatively higher adjusted means. No significant between-group differences were observed for the remaining measurements. **Conclusions:** Frontal sinus hypoplasia/aplasia, particularly unilateral and bilateral frontal sinus variation, was associated with selected differences in craniofacial morphology, especially in the orbital and upper facial regions, rather than demonstrating a direct effect. These findings may help to characterize craniofacial morphometric patterns in adults with sinus variation and could be considered in radiologic evaluation and preoperative assessment, but require confirmation in larger studies.

## 1. Introduction

The paranasal sinuses are closely related to adjacent structures and are of great importance in radiology, clinical medicine and surgical interventions, particularly in functional endoscopic sinus surgery [1]. They are air-filled cavities that form as a result of the expansion of the nasal cavities and erosion of adjacent bony structures [2]. The paranasal sinuses include the frontal, maxillary, ethmoid, and sphenoid sinuses [3]. Many studies have focused on the functions of the paranasal sinuses, such as sound resonance, humidification and air heating, thermal insulation, protection of the brain from trauma, lightening of the skull, and facial structure and growth. Proetz reported that human frontal and maxillary sinuses may have been designed to assist in the forward and downward growth of the face [3].

Developmentally, the paranasal sinuses form as evaginations of the rhinonasal mucosa during the fetal period and expand in volume with postnatal splanchnocranium growth. Although the ethmoid and maxillary sinuses are partially developed at birth, the frontal and sphenoid sinuses are absent in newborns and begin to develop later in childhood [4]. As the paranasal sinuses continue to develop throughout childhood, they are susceptible to significant anatomical variations and congenital malformations, as well as acquired infectious, inflammatory, and neoplastic diseases [5,6].

Understanding sinus development provides guidance in planning sinus surgery that can compromise sinus pneumatization and facial growth [7,8,9,10]. The pneumatization and development of the paranasal sinuses can show many different variations, and there are different rates of occurrence with different clinical effects. Among these variations, aplasia and hypoplasia are more common in the frontal sinuses than in the maxillary sinuses [11].

When incorporated into clinical practice, anthropometric measurements are important for measuring changes in the craniofacial framework. Anthropometric measurements for the analysis of facial anatomy include distance, relationship, and ratios [12]. It has been shown that there may be a connection between the volumetric/morphological characteristics of the sinuses and the general structure of the facial skeleton [13,14].

However, studies specifically examining the relationship between paranasal sinus hypoplasia/aplasia and facial morphometry remain limited. In this study, hypoplasia and aplasia were considered together within the broader category of sinus variation, as both reflect impaired sinus development and the available subgroup sizes were limited. To our knowledge, the association between frontal and maxillary sinus variations and CT-based facial morphometric measurements derived from anthropometric landmarks has not yet been clearly defined. Therefore, the aim of this study was to quantitatively evaluate facial morphometric differences and potential associations in adults with frontal and maxillary sinus hypoplasia/aplasia using standardized anthropometric reference points on CT images and to provide reference data for the literature.

## 2. Materials and Methods

This study was approved by the Ethics Committee of Antalya Training and Research Hospital (approval date: 11 July 2024; approval no: 10/14). Because of the retrospective design and the use of existing imaging and clinical records, the requirement for informed consent was waived by the Ethics Committee.

### 2.1. Study Design

The present study was designed as a retrospective case–control study. Adult patients who presented to the otolaryngology outpatient clinic and underwent paranasal sinus computed tomography (PNS CT) between 3 April 2023, and 30 May 2024, were retrospectively screened from the hospital records. CT images were reviewed jointly by an experienced otolaryngologist and radiologist. Patients meeting the inclusion and exclusion criteria and showing frontal or maxillary sinus hypoplasia/aplasia were included in the sinus variation groups. The control group consisted of adults who underwent paranasal sinus CT during the same study period, had no radiologic evidence of frontal or maxillary sinus hypoplasia/aplasia, and met the same exclusion criteria. Controls were not individually matched to cases by age or sex. The study population consisted of 117 patients with sinus variation (63 women and 54 men) and 53 healthy controls (21 women and 32 men).

Inclusion criteria were as follows: (1) adults aged 18 years and older, (2) patients with paranasal CT scans, and (3) patients with frontal or maxillary sinus variation. The exclusion criteria were as follows: (1) history of craniofacial syndrome, (2) endocrine diseases affecting the facial skeleton, (3) history of previous aesthetic or traumatic surgery in the facial region, (4) tumor in the paranasal sinuses, and (5) technical inadequacy affecting image quality. Information on prior facial trauma or previous facial/aesthetic surgery was screened from the documented medical history available in the electronic records associated with the CT examination. Only patients without a recorded history of facial trauma or prior facial/aesthetic surgery before CT acquisition were eligible for inclusion.

**Figure 1:**
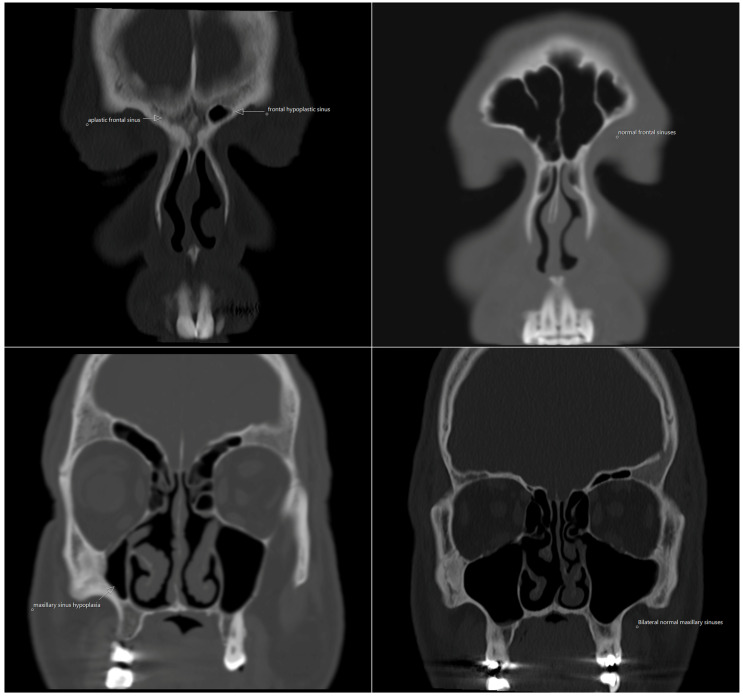
Upper photos, left side: frontal aplasia & hypoplasia, right side: normal frontal sinus. Lower photos, left side: maxillary sinus hypoplasia, right side: normal maxillary sinus.

We evaluated 3000 paranasal CT examinations obtained during the study period. Among these, all eligible adults with frontal and/or maxillary sinus hypoplasia/aplasia were included in the study. The sinus variation groups consisted of unilateral frontal sinus variation (UFSV), unilateral maxillary sinus variation (UMSV), bilateral frontal sinus variation (BFSV), and bilateral maxillary sinus variation (BMSV). Sinus variation groups included both hypoplastic and aplastic sinuses. These entities were analyzed together because both represent reduced or absent sinus development within the same anatomical region, and further subdivision into separate hypoplasia and aplasia subgroups would have resulted in very small subgroup sizes, particularly for maxillary sinus variation, limiting the robustness of statistical comparisons. The bilateral maxillary sinus variation (BMSV) subgroup consisted of only 3 cases. Because this number was too small for reliable inferential comparison, and because pooling bilateral and unilateral maxillary sinus variation would have introduced anatomical heterogeneity, the BMSV subgroup was described descriptively but was not included in the main statistical analyses. The healthy control group was formed from eligible adults from the same imaging pool who had no radiologic evidence of frontal or maxillary sinus hypoplasia/aplasia. The CT images obtained in this study are shown in Figure 1.

### 2.2. Imaging Protocol

A total of 3000 contrast-free paranasal sinus CT scans, acquired between 3 April 2023, and 30 May 2024, from the Picture Archiving and Communication Systems (PACS) Sectra PACS system of our institution, were examined. A CT 5300 (Philips Healthcare, Suzhou Co. Ltd., Suzhou, China) equipped with 64-row detectors and 128 slices was used for the CT imaging. The scan generated non-contrast continuous slices with characteristics including 1.0 mm slice thickness, 120 kV tube voltage, 200 mA tube current, and a field of view (FOV) of 140–160 mm. The scanning range encompassed the hard palate to the superior region of the frontal sinus. Subsequent coronal reconstruction was performed. The sections were reconstructed with a standard 512 × 512 matrix; for the 140–160 mm field of view, this corresponded to an in-plane pixel size of approximately 0.27–0.31 mm at a 1.0 mm reconstructed section thickness, so that the resulting voxels were not strictly isotropic. From this volumetric dataset, three-dimensional surface-rendered (volume-rendered) reconstructions were generated and used for all craniofacial measurements, while multiplanar reformatted images (axial, coronal, and sagittal planes) were available for anatomical orientation.

### 2.3. Image Analysis

Paranasal sinus CT images were reconstructed in three-dimensional format using 3D reconstruction on PACS. The reconstructions obtained were analyzed using the integrated measurement modules of the system, and linear distance measurements were performed between specific anthropometric reference points. All anatomical landmarks were identified and all linear craniofacial distances were measured directly on these three-dimensional surface-rendered (volume-rendered) reconstructions of the bony craniofacial framework, rather than on two-dimensional multiplanar reformatted images.

All craniofacial measurements were performed by two experienced anatomists using a consensus-based approach. Each CT image was examined jointly, and all anthropometric points were identified and measured by mutual agreement between the observers. The identification of each reference point and any discrepancies in the measurements were resolved by consensus until agreement was reached.

### 2.4. Measurements

The craniofacial distances measured were as follows: right and left orbital breadth, orbital height, interorbital breadth, biorbital breadth, minimum frontal breadth, upper facial breadth, bizygomatic breadth, nasal breadth, nasal aperture height, nasal height, and nasion–prosthion height. The definitions of these distances are given below.

1. Orbital Breadth: The distance from dacryon to ectoconchion.
2. Orbital Height: The distance between the superior and inferior orbital margins perpendicular to the orbital breadth, bisecting the orbit into equal medial and lateral halves.
3. lnterorbital Breadth: The distance between right and left dacryon.
4. Biorbital Breadth: The distance from left to right ectoconchion.
5. Minimum Frontal Breadth: The distance between the right and left frontotemporale.
6. Upper Facial Breadth: The distance between the right and left frontomalare temporale.
7. Bizygomatic Breadth: The distance between right and left zygion.
8. Nasal Breadth: The distance between right and left alare.
9. Nasal Aperture Height: The distance from rhinion to nasospinale.
10. Nasal Height: The distance from nasion to nasospinale.
11. Nasion–Prosthion Height: The distance from nasion to prosthion.

**Figure 2:**
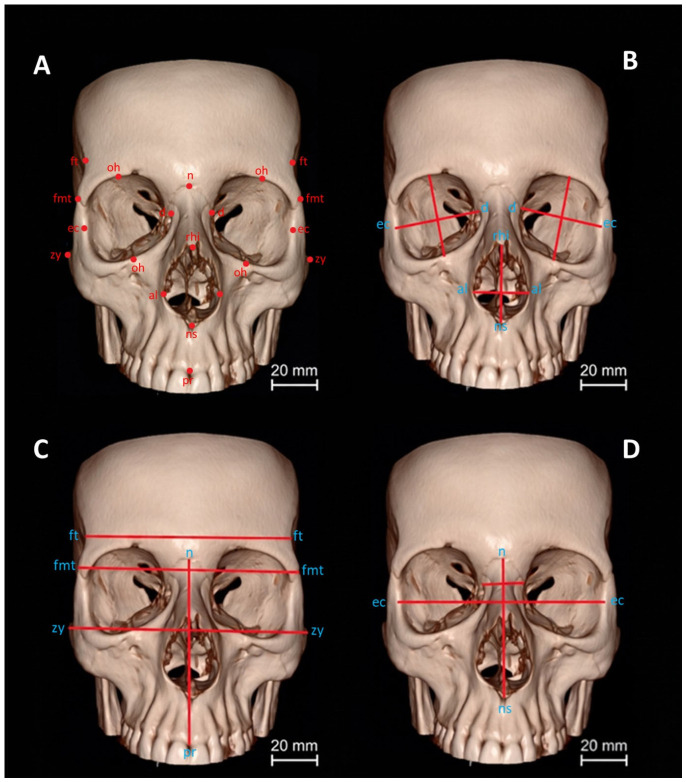
Measurements of craniofacial distances. (**A**), shows the craniofacial landmarks. (**B**–**D**), show illustrative example of measured distances. d-ec: orbital breadth, oh-oh: orbital height, al-al: nasal breadth, rhi-ns: nasal aperture height, ft-ft: minimum frontal breadth, fmt-fmt: upper facial breadth, zy-zy: bizygomatic breadth, n-pr: nasion–prosthion height, n-ns: nasal height, d-d: interorbital breadth, and ec-ec: biorbital breadth.

Craniofacial distances were determined using Data Collection Procedures for Forensic Skeletal Material 2.0 book [15]. The measurements are shown in Figure 2 and definitions of the craniofacial landmarks used for morphometric measurements are provided in Supplementary Table S1.

### 2.5. Statistical Analysis

Statistical analyses were performed using SAS version 9.4 statistical software package (Statistical Analysis Software SAS 9.4; SAS Institute, Cary, NC, USA). Means and standard deviations were used as descriptive statistics for quantitative variables, and numbers and percentages were used as descriptive statistics for qualitative variables. The Kolmogorov–Smirnov test and skewness coefficients were used to test whether the distribution of each quantitative variable was normally distributed. Skewness values between −2 and +2 are considered acceptable to prove a normal univariate distribution. The skewness values of each variable ranged from −2 to +2, and the results of Kolmogorov–Smirnov analyses showed a normal distribution; therefore, parametric statistical analyses were performed. Because controls were not individually matched to cases and the sex distribution differed across groups, age and sex were included as covariates in the ANCOVA models to reduce potential confounding. Analysis of covariance (ANCOVA) was used to compare group means in the craniofacial distances while controlling or adjusting for the influence of age and sex. Bonferroni corrected post hoc tests were used if a significant mean difference was found in the least-square group means. Bonferroni correction was applied across all multiple comparisons. The paired sample *t*-test was used for right-left comparisons of craniofacial distances.

Pearson correlation analysis was performed to reveal the association between craniofacial distance variables, and chi-square analysis was performed to reveal the relationship between qualitative variables. No a priori power or sample-size calculation was performed because this was a retrospective case–control study based on all eligible cases identified during the study period. Effect sizes (Cohen’s d) were reported to aid interpretation of the magnitude of group differences. Cohen’s d value was calculated to evaluate the effect size. An effect size (Cohen’s d) value was defined as small if it was less than 0.2, medium if it was 0.5, and large if it was greater than 0.8. Throughout the study, the threshold for statistical significance was set at *p* < 0.05.

## 3. Results

Our study included 117 patients with sinus variations and 53 healthy controls. The patients were divided into four groups. There were 46, 13, 55 in the UFSV, UMSV, BFSV group and three patients in the BMSV group, respectively. Among the patients in the UFSV group, 30 had right-sided sinus variations and 16 had left-sided sinus variations. Among the patients in the UMSV group, 11 had right-sided sinus variations and 2 had left-sided sinus variations. The BMSV subgroup included only 3 patients and was therefore not entered into the main comparative statistical analyses; these cases were considered descriptively only. The demographic characteristics of the study groups are presented in Table 1.

Analysis of covariance with the subject’s sex and age as covariates was performed to compare groups for each of the craniofacial distances; the results are presented in Table 2. Unadjusted means and standard errors for all craniofacial distances in different groups are presented in Supplementary Table S2.

When the least square mean values of the measured parameters were examined, the left orbital breadth, right orbital breadth, and biorbital breadth were found to be the highest in the control group, whereas the left orbital height, upper facial breadth, and bizygomatic breadth were highest in the UMSV group. On the other hand, the least square mean values of the left orbital height, biorbital breadth, upper facial breadth, and bizygomatic breadth were lowest in the BFSV group, and the left orbital breadth and right orbital breadth had the lowest least square mean values in the UFSV group. The least square mean values of orbital height, interorbital breadth, minimum frontal breadth, nasal breadth, nasal aperture height, nasal height, and nasion–prosthion height were similar between the groups (*p* > 0.05).

In this analysis, statistically significant differences were found between the least squares means of the groups in the left orbital breadth (*p* = 0.0001), left orbital height (*p* = 0.0250), right orbital breadth (*p* = 0.0000), biorbital breadth (*p* = 0.0000), upper facial breadth (*p* = 0.0204), and bizygomatic breadth (*p* = 0.0026). In all other measured variables (right orbital height, orbital breadth, minimum frontal breadth, nasal breadth, nasal aperture height, nasal height, and nasion–prosthion height), no statistically significant differences were found between the groups (*p* > 0.05).

For all the significant craniofacial distances, Bonferroni-corrected pairwise comparisons showed that there were no significant differences between the least square means of HC and UMSV and of BFSV and UFSV, and the least square means of HC and UMSV subjects were significantly higher then those of BFSV and UFSV patients.

The right and left orbital measurements of the groups with unilateral sinus variation according to the variation side were compared, and the results are presented in Table 3.

As can be seen from this table, no statistically significant differences were found between the right and left sides in orbital breadth and height measurements for both the UFSV and UMSV groups. Similarly, no statistically significant differences were observed in the right and left orbital breadth and orbital height measurements in the BFSV group. However, in the HC group, however, the right orbital height measurements were significantly lower than those on the left side (*p* = 0.0051).

**Figure 3:**
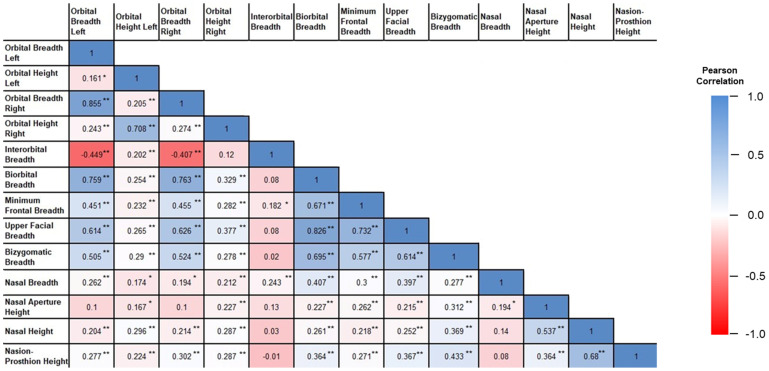
Correlation between craniofacial distances. * Correlation is significant at the 0.05 level. ** Correlation is significant at the 0.01 level.

Analysis of the relationships among the craniofacial parameters revealed that the majority of craniofacial dimensions were significantly correlated. When the parameters were compared, the following pairs demonstrated a very strong positive correlation: right orbital breadth with left orbital breadth, right orbital height with left orbital height, both orbital breadths with biorbital breadth, upper facial breadth with biorbital breadth, and upper facial breadth with minimum frontal breadth. In contrast, both orbital breadths showed a moderate negative correlation with the interorbital breadth. The results of the Pearson correlation analysis for the 13 measured craniofacial distances are shown in Figure 3. The complete correlation matrix, including all correlation coefficients and corresponding *p* values are presented in Supplementary Table S3.

## 4. Discussion

Preoperative endoscopic evaluation of sinus anatomy can be performed using CT, which is the gold standard, providing information about bone structures and air, thus providing a highly sensitive imaging method [2].

The frontal sinus is the most complex of the paranasal sinuses owing to its location, anatomical variations, and multiple clinical presentations. Frontal sinuses are not present at birth and are usually well developed during childhood. They continue to grow gradually until reaching their maximum size after puberty [16].

The maxillary sinuses are the largest among the paranasal sinuses. The maxillary sinus has a significant impact on the diagnosis and semiology of craniofacial structures [17]. Maxillary sinus hypoplasia is less common than sphenoid or frontal sinus hypoplasia, and may be acquired or congenital. Uncinate process abnormalities associated with maxillary sinus hypoplasia may inadvertently cause orbital complications during surgery [18].

The development of the paranasal sinuses is closely related to the growth of the facial skeleton and is an important factor to be considered in surgical planning [10]. Additionally, many studies have shown that growth of the maxilla and nasal cavities is closely related to the development of the sinuses, and these structures ultimately determine the morphology of the face [19].

Although the possible relationships between frontal and maxillary sinus variations and facial morphology have been reported in the literature, to our knowledge, no studies have specifically evaluated how these variations are associated with facial morphometry.

In the current study, we analyzed the differences in facial morphometry at some anthropometric points between individuals with sinus variations and individuals with healthy sinuses.

Our findings suggest that frontal sinus hypoplasia/aplasia may be associated with selected differences in craniofacial measurements, particularly in the orbital region.

Because of the retrospective and cross-sectional nature of this study, the observed relationships should be interpreted as associations rather than evidence of a developmental causal effect. It is possible that frontal sinus hypoplasia/aplasia and the measured craniofacial features reflect shared developmental determinants rather than a direct influence of one structure on the other. Alternative explanations include common genetic or embryologic factors affecting craniofacial growth, as well as measurement variability or selection-related bias inherent to retrospective imaging-based studies.

In our study, when we compared the HC group with other groups, we found that left orbital breadth, left orbital height, right orbital breadth, biorbital breadth, upper facial breadth, and bizygomatic breadth were significantly lower in the UFSV and BFSV groups than in the HC group. There was no statistically significant difference between the HC and UMSV groups. In the comparison of each parameter, the measurements related to the orbit and upper face region generally showed a very strong positive correlation with each other.

Aslier et al. investigated the effects of craniofacial structure and nasal septum deviation on frontal sinus morphology in 3 D using 74 dry skulls. As a result of their craniofacial measurements, they found the minimum frontal breadth as 94.42 ± 4.66 mm, bizygomatic breadth as 125.88 ± 6.92 mm, nasal breadth as 23.90 ± 2.05 mm, and nasal height as 50.12 ± 3.82 mm. In our study, we found the minimum frontal breadth as 96.58 ± 0.68 mm, bizygomatic breadth as 125.71 ± 0.89 mm, nasal breadth as 23.89 ± 0.28 mm, and nasal height as 53.29 ± 0.55 mm in healthy individuals. In the UFSV group, we found the minimum frontal breadth as 97.48 ± 0.72 mm, bizygomatic breadth as 124.36 ± 0.95 mm, nasal breadth as 23.65 ± 0.30 mm, and nasal height as 53.61 ± 0.59 mm. In the BFSV group, we found the minimum frontal breadth as 96.90 ± 0.67 mm, bizygomatic breadth as 123.87 ± 0.88 mm, nasal breadth as 23.23 ± 0.28 mm, and nasal height as 53.24 ± 0.55 mm. Accordingly, we observed that nasal breadth was not affected by sinus variations. In addition, bizygomatic breadth showed a significant decrease especially in the UFSV and BFSV groups, suggesting that sinus variations may reflect shared developmental patterns.

Aslier et al. investigated the relationship between different pneumatized frontal sinuses and cranial variables in groups with craniofacial and bony nasal septum deviation, and found a relationship between maxillary sinus breadth and nasal height in the right frontal sinus group, maximum head length, upper face height, and nasal height in the left frontal sinus group, zygomatic breadth, and nasal height. These studies have reported associations between sinus dimensions and facial height; however, such findings do not establish causality, and similar relationships may reflect shared developmental or genetic influences [14]. In our study, we found no difference in nasal height between the control and frontal sinus groups with varied sinuses.

Abeta et al. investigated the relationship between morphometric measurements of the frontal sinuses and facial growth patterns by selecting individuals who had undergone 80-cone beam computed tomography (CBCT) scans. To evaluate the relationship between the craniofacial features and frontal sinus characteristics, cephalometric measurements were performed for each individual. They demonstrated a correlation between frontal sinus dimensions and craniofacial features [17].

Gursoy et al. evaluated three-dimensional frontal sinus morphology, taking into account different vertical facial developments, and reported that the anteroposterior dimension of the frontal sinuses decreased in individuals with a vertical growth pattern and that significant correlations emerged with vertical craniofacial parameters [20]. Furthermore, Albitar et al. evaluated facial measurements in relation to facial aesthetics in patients with a vertical growth pattern. In their study, they found that among individuals with a vertical growth pattern, lower mouth width/face height ratios in women and increased facial convexity angles in men were associated with higher attractiveness, and these findings highlighted the importance of including these variables in orthodontic diagnosis and treatment planning to improve facial aesthetics. Although our study focused on skeletal craniofacial distances rather than aesthetic evaluation, these findings suggest that evaluating facial morphometry in individuals with different craniofacial patterns can provide valuable clinical and anatomical information [21].

Oksayan et al. divided 60 adults equally into three groups (high-angle, low-angle, and normal-angle groups) according to skeletal vertical facial growth patterns and compared maxillary sinus volume and dimensions using CBCT with cephalometric measurements. The researchers’ findings indicated that subjects with a high angle exhibited statistically lower values with regard to maxillary sinus length and breadth than subjects with a low angle. This study demonstrated that vertical facial growth had no impact on the right and left maxillary sinus volumes [13]. In addition, Azizia et al. evaluated the relationship between sagittal skeletal discrepancies and maxillary sinus volume in adults using CBCT. Their study found no significant difference in maxillary sinus volume, surface area, and height among skeletal classes; however, they reported a significant increase in maxillary sinus width and depth in skeletal class I compared to skeletal class III [22].

In the present study, we investigated the relationship between craniofacial distances in individuals with sinus variation and healthy individuals. The investigation revealed a positive correlation between the transverse craniofacial distances and a negative correlation between the vertical distances.

In summary, we observed no clear morphological impact of sinus variations on facial symmetry. These variations may affect more bone structures but may not change the general facial structure. However, the presence of similar variations may lead to more pronounced morphological changes in individuals during the growth period, suggesting that the observed associations may vary across developmental stages; however, the present adult cross-sectional dataset does not allow conclusions regarding growth-related effects over time.

From an otolaryngological perspective, these anatomical variants may be relevant during radiologic evaluation and preoperative assessment; however, their clinical implications require confirmation in larger studies. Although several group differences reached statistical significance, the absolute magnitude of these differences was small and measured in millimetres. Therefore, these findings should be interpreted primarily as morphometric associations at the group level rather than as differences with clear standalone clinical significance for individual patients. In particular, the observed differences are unlikely to directly change radiologic interpretation or surgical decision-making on their own, but they may contribute to a broader anatomical understanding of craniofacial patterns associated with sinus variation. In this context, awareness of such patterns may still be useful during preoperative imaging review, particularly in patients undergoing endoscopic sinus surgery or procedures involving the orbit or anterior skull base. However, these morphometric differences should be regarded as adjunctive anatomical information rather than independent determinants of surgical strategy.

The cranial base is at the intersection of many sub disciplines because its proximity to the orbit and maxilla. Therefore, it is important to understand the morphology of the frontal and maxillary sinuses, which are anatomical structures.

Frontal sinuses sometimes become excessively enlarged, but in pneumatosel types, they have been reported to cause facial asymmetry and changes in facial contours [23].

Overly wide frontal sinuses can be observed together with facial asymmetry and can cause serious aesthetic problems. Forehead and frontal sinus surgeries may be necessary for some rare craniofacial malformations, such as pneumosinus dilatans. In such patients, the aim is to correct the protruding forehead/frontal boss by minimizing the overly wide frontal sinuses [24].

Surgeons sometimes enter the skull through the frontal sinus, and awareness of the asymmetrical frontal sinuses is very important in minimizing surgical complications. In frontal sinus surgery, especially in patients with narrow or medium-breadth head/upper face structures and asymmetric skull base openings, detailed anatomical knowledge and preoperative evaluation with CT are of great importance because of the frequent occurrence of anatomical variations and proximity to the orbit and anterior skull base.

We believe this study may contribute to the anatomical characterization of craniofacial morphometric patterns associated with sinus variation. However, the observed differences were small in absolute magnitude and should be interpreted cautiously with respect to direct clinical application.

This study has several limitations. First, its retrospective design does not allow causal inference and may be subject to incomplete or unevenly documented clinical information. Many factors known to influence craniofacial morphology and dimensions—such as ethnic origin, body measurements (height and weight), dental condition, history of previous orthodontic or orthognathic treatment, and skeletal classification—were not available in the database and, due to the retrospective nature of this study, could not be systematically recorded. Consequently, in the statistical analyses, only age and sex were controlled for using ANCOVA as covariates. Consequently, these variables may have a potential effect on craniofacial measurements. Second, the study was conducted at a single center, which may limit the representativeness of the sample. Third, because participants were selected from patients who underwent paranasal CT in a clinical setting, selection bias cannot be excluded, and the findings may therefore have limited generalizability beyond similar adult hospital-based populations. In addition, the sex distribution of the groups was not homogeneous. To reduce potential confounding, age and sex were included as covariates in the ANCOVA models; however, residual confounding cannot be excluded. Controls were selected from eligible individuals imaged during the same study period rather than being individually matched to cases. Furthermore, no a priori power or sample-size calculation was performed because the study was based on retrospectively available eligible cases and controls. Another limitation is that hypoplasia and aplasia were analyzed together within the same variation groups. Although these entities are biologically distinct, separate subgroup analyses were not feasible because of the limited number of cases in several subgroups, particularly those involving maxillary sinus variation. Lastly, all measurements were obtained through a consensus-based assessment carried out jointly by two experienced anatomists. Although this approach aimed to minimise variability in the determination of reference points and measurement procedures, as the measurements were not recorded independently, formal inter-observer and intra-observer reliability analyses, such as intraclass correlation coefficient (ICC) calculations or Bland–Altman plots, could not be performed.

Future studies evaluating morphometric variables in facial asymmetry with a larger number of subjects are necessary to confirm our findings. Furthermore, we believe that the findings of this study can serve as a starting point for future studies that could advance our understanding of facial morphology development and its contribution to the presence of sinus variations and healthy controls.

In conclusion, individuals with unilateral and bilateral frontal sinus variation showed significantly lower left orbital breadth, right orbital breadth, and biorbital breadth compared with healthy controls, whereas no significant differences were observed for most other measurements or in the maxillary sinus variation groups. These findings indicate an association between frontal sinus hypoplasia/aplasia and selected craniofacial morphometric differences, particularly in the orbital region. However, the present study does not support causal inference, and the observed relationships may also reflect shared developmental factors or methodological limitations. Further studies with larger and more balanced samples are needed to clarify the biological and clinical relevance of these associations.

## Figures and Tables

**Table 1:** Demographic characteristics of study groups.

	HC (*N* = 53)	UFSV (*N* = 46)	UMSV (*N* = 13)	BFSV (*N* = 55)	*p*-Value
Sex, *n* (%)					**0.0149** **^1^**
Female	21 (39.6%)	25 (54.3%)	3 (23.1%)	35 (63.6%)	
Male	32 (60.4%)	21 (45.7%)	10 (76.9%)	20 (36.4%)	
Age					0.3412 ^2^
Mean (SD)	40.3 (15.84)	39.2 (17.86)	44.2 (16.33)	45.0 (14.35)	
Median (Range)	39.0 (18.0, 75.0)	39.0 (11.0, 78.0)	46.0 (17.0, 70.0)	46.0 (18.0, 72.0)	

Bold values denote statistically significant differences between groups (*p* < 0.05). ^1^ Chi-square test; ^2^ One-way ANOVA. HC, healthy controls; UFSV, unilateral frontal sinus variation; UMSV, unilateral maxillary sinus variation; BFSV, bilateral frontal sinus variation; SD, standard deviation.

**Table 2:** Gender and age-adjusted least square means, standard errors and effect sizes (Cohen’s d) for all craniofacial distances (mm) in different groups (BFSV, UFSV, HC and UMSV).

Craniofacial Distances	BFSV		UFSV		HC		UMSV		*p * ^1^	Cohen’s d (95% CI)
	LS Mean	StdErr	LS Mean	StdErr	LS Mean	StdErr	LS Mean	StdErr		
Orbital Breadth-Left	39.64 b	0.36	39.19 b	0.39	40.87 a	0.37	40.68 a	0.74	**0.0001**	0.73 (0.36, 1.01)
Orbital Height-Left	34.54 b	0.36	34.89 b	0.38	35.67 a	0.36	36.34 a	0.73	**0.0250**	0.49 (0.01, 0.75)
Orbital Breadth-Right	39.67 b	0.35	39.08 b	0.38	40.98 a	0.35	40.87 a	0.71	**0.0000**	0.81 (0.44, 1.09)
Orbital Height-Right	34.62	0.34	34.48	0.36	34.88	0.34	36.15	0.69	0.0952	0.40 (0.00, 0.65)
Interorbital Breadth	20.91	0.42	22.15	0.45	21.54	0.42	21.23	0.86	0.5709	0.22 (0.00, 0.45)
Biorbital Breadth	95.53 b	0.55	95.68 b	0.59	98.07 a	0.55	97.72 a	1.12	**0.0000**	0.81 (0.44, 1.09)
Minimum Frontal Breadth	96.90	0.67	97.48	0.72	96.58	0.68	96.76	1.37	0.8792	0.13 (0.00, 0.29)
Upper Facial Breadth	102.89 b	0.67	103.13 b	0.72	104.02 a	0.68	105.13 a	1.37	**0.0204**	0.50 (0.06, 0.76)
Bizygomatic Breadth	123.87 b	0.88	124.36 b	0.95	125.71 a	0.89	126.66 a	1.80	**0.0026**	0.61 (0.22, 0.87)
Nasal Breadth	23.23	0.28	23.65	0.30	23.89	0.28	24.04	0.57	0.1210	0.38 (0.00, 0.63)
Nasal Aperture Height	34.71	0.47	35.48	0.51	33.72	0.47	34.32	0.96	0.2355	0.33 (0.00, 0.57)
Nasal Height	53.24	0.55	53.61	0.59	53.29	0.55	52.65	1.12	0.3492	0.29 (0.00, 0.52)
Nasion–Prosthion Height	70.57	0.86	72.10	0.93	71.11	0.87	71.68	1.76	0.0817	0.41 (0.00, 0.66)

Bold values denote statistically significant differences (p < 0.05). ^1^ ANCOVA F-test. Values sharing the same lowercase letter within a row were not significantly different in Bonferroni-corrected pairwise comparisons. UFSV = unilateral frontal sinus variation; UMSV = unilateral maxillary sinus variation; BFSV = bilateral frontal sinus variation; HC = healthy control.

**Table 3:** Comparison of orbital measurements of UFSV and UMSV groups (mm).

			Left Side			Right Side			*p*	Cohen’s d (95% CI)
			*N*	Mean	Sd	*N*	Mean	Sd		
Right sinus variation	UFSV	Orbital Breadth	30	39.33	2.67	30	39.38	2.54	0.8606	0.032 (−0.39, 0.33)
		Orbital Height	30	34.86	2.53	30	34.62	2.12	0.4895	0.128 (−0.23, 0.49)
	UMSV	Orbital Breadth	11	41.50	2.41	11	41.47	2.14	0.9474	0.020 (−0.57, 0.61)
		Orbital Height	11	35.81	1.53	11	35.79	1.58	0.9455	0.021 (−057, 0.61)
Left sinus variation	UFSV	Orbital Breadth	16	38.76	2.27	16	38.42	2.75	0.3523	0.240 (−0.26, 0.73)
		Orbital Height	16	34.75	2.61	16	34.07	1.70	0.1411	0.388 (−0.13, 0.89)
	UMSV	Orbital Breadth	2	39.95	0.78	2	40.85	0.07	0.3228	1.273 (0.83, 3.26)
		Orbital Height	2	40.30	6.08	2	39.15	1.77	0.8699	0.147 (−1.28, 1.51)
Bilateral sinus variation	BFSV	Orbital Breadth	55	39.29	2.35	55	39.33	2.24	0.8456	0.026 (−0.29, 0.24)
		Orbital Height	55	34.53	2.62	55	34.58	2.90	0.8644	0.023 (−0.29, 0.24)
	HC	Orbital Breadth	53	41.12	3.61	53	41.23	3.39	0.6891	0.055 (−0.32, 0.21)
		Orbital Height	53	35.69	2.62	53	34.92	2.45	**0.0051**	0.402 (0.12, 0.68)

Bold values indicate statistically significant differences (p < 0.05). UFSV = unilateral frontal sinus variation; UMSV = unilateral maxillary sinus variation; BFSV = bilateral frontal sinus variation; HC = healthy control.

## Data Availability

The data presented in this study are available on reasonable request from the corresponding author. The data are not publicly available due to privacy restrictions and ethical considerations regarding patient confidentiality.

## Supplementary Materials

The following supporting information can be downloaded at: https://www.mdpi.com/article/10.3390/diagnostics16132056/s1, Table S1: Definitions of craniofacial landmarks used for morphometric measurements. Table S2. Unadjusted means and standard errors for all craniofacial distances (mm) in different groups (BFSV, UFSV, HC AND UMSV). Table S3. Correlation coefficients (r) and corresponding *p*-values for all pairwise associations between morphometric measurements.
