# Supplementary material for: CraniofacialMorphometric Associations with Frontal Sinus Hypoplasia/Aplasia in Adults: Orbital and Upper Facial Differences on CT
Source: Diagnostics (Basel). 2026 Jun 30;16(13):2056. doi: 10.3390/diagnostics16132056 (PMC13359954; doi:10.3390/diagnostics16132056)
Supplement: Supplementary file 1 [file diagnostics-16-02056-s001.zip › diagnostics-4326263-supplementary.pdf]

Table S1. Definitions of craniofacial landmarks used for morphometric measurements.

Landmark	Full Name	Definition
d	Dacryon	The point of intersection of the frontal, lacrimal, and maxillary bones at the medial orbital margin.
ec	Ectoconchion	The most lateral point on the orbital margin.
ft	Frontotemporale	A point located forward and inward, above the superior temporal line, just above the zygomatic process of the frontal bone.
fmt	Frontomalare temporale	The most lateral point of the fronto-malar suture.
n	Nasion	The midpoint of the fronto-nasal suture in the midsagittal plane.
pr	Prosthion	The most anterior midline point on the alveolar process of the maxilla between the upper central incisors
zy	Zygion	The maximum breadth across the zygomatic arches, wherever, perpendicular to the mid-sagittal plane.
al	Alare	The most lateral point on the margin of the nasal aperture.
rhi	Rhinion	The inferior endpoint of the internasal suture in the midsagittal plane.
ns	Nasospinale	The point where a line connecting the inferior margins of the nasal aperture intersects the midsagittal plane.

Table S2. Unadjusted means and standard errors for all craniofacial distances (mm) in different groups (BFSV, UFSV, HC AND UMSV)

	**BFSV**		**UFSV**		**HC**		**UMSV**		
**Craniofacial distances**	**Mean**	**StdErr**	**Mean**	**StdErr**	**Mean**	**StdErr**	**Mean**	**StdErr**	**P[1]**
Orbital Breadth-Left	39.29	0.32	39.13	0.37	41.12	0.50	41.26	0.63	**0.0111**
Orbital Height-Left	34.53	0.35	34.82	0.37	35.69	0.36	36.50	0.78	**0.0541**
Orbital Breadth-Right	39.33	0.30	39.05	0.39	41.23	0.47	41.38	0.55	**0.0017**
Orbital Height-Right	34.58	0.39	34.43	0.29	34.92	0.34	36.31	0.55	0.1886
Interorbital Breadth	21.15	0.39	22.00	0.39	21.41	0.55	21.29	0.74	0.2527
Biorbital Breadth	95.13	0.50	95.41	0.68	98.42	0.67	98.85	1.31	**0.0040**
Minimum Frontal Breadth	96.64	0.59	97.24	0.68	96.81	0.81	97.63	1.73	0.8366
Upper Facial Breadth	102.43	0.81	102.82	0.68	104.41	0.73	106.44	1.39	0.4011
Bizygomatic Breadth	122.95	0.86	123.82	1.17	126.47	1.16	129.05	2.19	0.3523
Nasal Breadth	23.25	0.25	23.45	0.32	23.93	0.38	24.49	0.48	0.3468
Nasal Aperture Height	34.29	0.45	35.31	0.59	34.05	0.53	35.25	1.20	0.0874
Nasal Height	52.55	0.56	53.36	0.87	53.82	0.60	54.08	0.91	0.8906
Nasion-Prosthion Height	69.55	0.64	71.78	1.38	71.89	1.03	73.69	1.66	0.3658

[1] ANCOVA F-test unadjusted p-value; Same superscript lowercase letters represent no significant difference in rows. UFSV: Unilateral Frontal Sinus Variation... UMSV: Unilateral Maxillary Sinus Variation. BFSV: Bilateral Frontal Sinus Variation. HC: Healthy control.

Table S3. Correlation coefficients (r) and corresponding p-values for all pairwise associations between morphometric measurements

	Orbital Breadth-Left	Orbital Height-Left	Orbital Breadth-Right	Orbital Height-Right	Interorbital Breadth	Biorbital Breadth	Minimum Frontal Breadth	Upper Facial Breadth	Bizygomatic Breadth	Nasal Breadth	Nasal Aperture Height	Nasal Height	Nasion-Prosthion Height
Orbital Breadth-Left	1.0000												
Orbital Height-Left	0.1614<br>0.0354	1.0000											
Orbital Breadth-Right	0.8549<br><.0001	0.2052<br>0.0072	1.0000										
Orbital Height-Right	0.2430<br>0.0014	0.7079<br><.0001	0.2744<br>0.0003	1.0000									
Interorbital Breadth	-0.4494<br><.0001	0.2016<br>0.0084	-0.4068<br><.0001	0.1183<br>0.1242	1.0000								
Biorbital Breadth	0.7586<br><.0001	0.2544<br>0.0008	0.7626<br><.0001	0.3286<br><.0001	0.0755<br>0.3274	1.0000							
Minimum Frontal Breadth	0.4508<br><.0001	0.2318<br>0.0023	0.4549<br><.0001	0.2817<br>0.0002	0.1815<br>0.0178	0.6711<br><.0001	1.0000						
Upper Facial Breadth	0.6142<br><.0001	0.2646<br>0.0005	0.6261<br><.0001	0.3768<br><.0001	0.0835<br>0.2789	0.8257<br><.0001	0.7321<br><.0001	1.0000					
Bizygomatic Breadth	0.5052<br><.0001	0.2900<br>0.0001	0.5237<br><.0001	0.2775<br>0.0002	0.0243<br>0.7526	0.6948<br><.0001	0.5773<br><.0001	0.6141<br><.0001	1.0000				
Nasal Breadth	0.2621<br>0.0006	0.1742<br>0.0231	0.1942<br>0.0111	0.2115<br>0.0056	0.2425<br>0.0014	0.4066<br><.0001	0.3003<br><.0001	0.3973<br><.0001	0.2773<br>0.0003	1.0000			
Nasal Aperture Height	0.0992<br>0.1978	0.1672<br>0.0292	0.0967<br>0.2094	0.2274<br>0.0029	0.1309<br>0.0888	0.2268<br>0.0029	0.2624<br>0.0005	0.2146<br>0.0049	0.3119<br><.0001	0.1943<br>0.0111	1.0000		
Nasal Height	0.2038<br>0.0077	0.2959<br><.0001	0.2142<br>0.0050	0.2869<br>0.0001	0.0273<br>0.7238	0.2613<br>0.0006	0.2184<br>0.0042	0.2519<br>0.0009	0.3688<br><.0001	0.1417<br>0.0652	0.5370<br><.0001	1.0000	
Nasion-Prosthion Height	0.2773<br>0.0003	0.2242<br>0.0033	0.3020<br><.0001	0.2869<br>0.0001	-0.0138<br>0.8579	0.3639<br><.0001	0.2713<br>0.0003	0.3674<br><.0001	0.4329<br><.0001	0.0830<br>0.2815	0.3638<br><.0001	0.6796<br><.0001	1.0000
